# Disseminated histoplasmosis and presumptive CNS toxoplasmosis-associated immune reconstitution inflammatory syndrome in a patient with HIV/AIDS: a case report

**DOI:** 10.1186/s12879-024-10262-x

**Published:** 2024-12-04

**Authors:** Nicolás Laverde-Sudupe, Erin R. Carr, Bruno Velit-Rios, Maria Morel-Almonte, Jose Guillermo Castro

**Affiliations:** 1 Pontificia Universidad Javeriana de Cali, Valle del Cauca, Cali, Colombia; 2 University of Miami Miller School of Medicine, Miami, FL USA; 3 Universidad Científica del Sur, Lima, Peru; 4 Division of Infectious Diseases, Leonard M. Miller School of Medicine, University of Miami, Miami, FL USA

**Keywords:** HIV, Histoplasma, Immune reconstitution inflammatory syndrome, Toxoplasmosis

## Abstract

**Background:**

Co-infections associated with Immune Reconstitution Inflammatory Syndrome (IRIS) have been described in literature, however they constitute an uncommon finding in the medical community.

**Case presentation:**

Here we report a rare case of a 55-year-old woman from Cuba with prior medical history of HIV/AIDS adherent to her antiretroviral therapy (ART) regimen, who was hospitalized in Miami, Florida because of fluid dysphagia, odynophagia and right-sided cervical lymphadenopathy. A prior biopsy of the right cervical lymph node performed in an outside hospital found evidence of non-caseating granulomas with budding yeast, which was later confirmed to be disseminated histoplasmosis by a positive (1-3) -β-glucan assay and histoplasmosis urine antigen in this admission. Furthermore, after multiple imaging testing due to her clinical condition, a brain MRI demonstrated findings concerning for cerebral toxoplasmosis, which was supported by serology findings. Treatment with liposomal amphotericin B and TMP-SMX led to clinical and radiological improvement of this patient’s conditions, and she was discharged with an appointment for follow-up in the clinic.

**Conclusion:**

This case highlights the complexities and challenges in managing opportunistic infections (OIs) during immune recovery in HIV/AIDS patients on ART, and emphasizes the necessity of continuous, vigilant monitoring and having a broad differential diagnosis in this group of patients.

## Background

Antiretroviral therapy (ART) has revolutionized the management of HIV/AIDS by significantly reducing viral load and improving CD4 counts, leading to a marked decrease in the incidence of opportunistic infections (OIs) [1]. However, approximately 10–32% of patients initiated on ART may experience a paradoxical clinical deterioration evidenced by either worsening or development of new symptoms or radiologic findings–a phenomenon known as Immune Reconstitution Inflammatory Syndrome (IRIS) [2, 3]. IRIS typically arises within three months of starting ART, though it can manifest later, especially in response to non-viable pathogen antigens [2]. This syndrome can be triggered by the immune system’s inflammatory response to both active infections and residual antigens from previously controlled infections [2].

Risk factors for developing IRIS include a low baseline CD4 count (< 100 cells/µL), a rapid increase in CD4 count following the initiation of ART, rapid suppression of HIV RNA, and the presence of latent OIs with a high antigenic burden [3]. The pathogenesis of IRIS is complex and varies with the specific pathogen involved. Typically, it is associated with a CD4 Th1-mediated immune response, though both CD4 and CD8 cells play crucial roles in the imbalanced immune response observed in IRIS [3].

A broad spectrum of organisms have been reported to trigger IRIS [4]. Though less frequently associated, *Histoplasma* species (spp.) and *Toxoplasma gondii* (*T. gondii)* have been reported in IRIS cases. Specifically, the incidence of IRIS related to *Histoplasma* is estimated to be 0.74 per 1,000 HIV-infected person-years, highlighting its rarity [5]. Additionally, IRIS associated with *T. gondii* has been extremely rare, with only six cases documented up to 2017 [6].

The combination of disseminated histoplasmosis and presumptive cerebral toxoplasmosis in the context of IRIS is exceptionally rare and has only been reported once in the medical literature [7]. To our knowledge, this case is likely the first documented report in the United States (U.S.) of a patient presenting with disseminated histoplasmosis-associated IRIS and cerebral toxoplasmosis co-infection.

## Case presentation

A 55-year-old Cuban female, diagnosed with HIV/AIDS six months prior, presented to the emergency department due to a four-month history of fluid dysphagia, a lump on the right side of her neck, and one month history of odynophagia. She reported adherence to her ART regimen of bictegravir/emtricitabine/tenofovir alafenamide (B/FTC/TAF) for the past three months. Of note, the patient immigrated to Miami, Florida from Cuba in December 2023 and denied any other recent travel.

The patient had recently been admitted to another hospital for similar symptoms associated with subjective fevers, chills, significant weight loss, and fatigue. Her CD4 count at the time was 4 cells/mm^3^. Due to her nonspecific symptoms, she was evaluated for tuberculosis and the diagnosis was excluded with negative acid-fast bacilli (AFB) stains on both sputum and bronchoalveolar lavage (BAL). She underwent a biopsy of the right cervical lymph node, which revealed non-caseating granulomas with budding yeast. However, this finding was not further investigated, and the patient was discharged before the fungal organism could be identified. She was diagnosed with esophageal candidiasis based on physical examination findings of multiple white plaques on her palate and tongue. She was treated with intravenous (IV) fluconazole 200 mg while inpatient, and discharged on itraconazole 200 mg daily for 4 weeks. She was also discharged on her B/FTC/TAF and prophylactic antimicrobials including trimethoprim-sulfamethoxazole (TMP-SMX) (800/160 mg daily) and azithromycin (1200 weekly) for 4 weeks.

On arrival at our facility, the patient was afebrile, reported persistent odynophagia, and worsening of her constitutional symptoms described above. The physical examination of the oral cavity again revealed multiple white plaques on her palate and tongue, consistent with candidiasis. She received fluconazole 400 mg IV once in the emergency department and was transitioned to fluconazole 200 mg orally. She had palpable, painful lymphadenopathy of the right cervical lymph nodes. No hepatosplenomegaly was appreciated. The patient had no focal neurological deficits. Laboratory findings were significant for a white blood cell count (WBC) of 6600/µl, hemoglobin of 12.5 g/dL, and platelet count of 307 × 10^4^ /µl. CD4 count was 68 cell/mm^3^ and viral load was undetectable.

Several imaging studies were obtained via pan-scan. An initial chest X-ray showed diffuse infiltrates on the pulmonary parenchyma, which warranted further imaging. CT scan of her neck and chest showed evidence of innumerable pulmonary nodules, extensive mediastinal lymphadenopathy, and an irregular enhancing lesion in the region of the left head of the caudate nucleus (Image 1). She underwent brain MRI which found a 1.4 cm ring-enhancing lesion surrounded by edema and probable hemorrhagic components, concerning for cerebral toxoplasmosis (Image 2). Based on the imaging findings, *Toxoplasma* serologies were obtained. *Toxoplasma* IgG resulted positive and IgM was negative. She was treated with IV TMP 5 mg/kg and SMX 25 mg/kg for cerebral toxoplasmosis. Abdominal CT showed numerous necrotic lymphadenopathy.

An extensive workup was undertaken in collaboration with infectious disease specialists. Upon reviewing the medical records and the cervical lymph node biopsy from admission at the outside hospital, a [1–3]-β-glucan assay was ordered and resulted positive at 384 pg/mL. Although a histoplasmosis urine antigen test was ordered at the outside hospital and returned negative at the time, it was repeated at our facility due to a high suspicion for disseminated histoplasmosis. The repeat histoplasmosis urine antigen resulted positive at > 15 ng/mL (reference range < 0.2 ng/mL). Blood cultures were obtained and showed no growth to date at 96 h. Given the patient’s history and clinical deterioration, evidenced by the development of constitutional symptoms, lymphadenopathy, and both laboratory and radiological findings following initiation of ART, a diagnosis of disseminated histoplasmosis-associated IRIS was made. Treatment with liposomal amphotericin B IV 5 mg/kg was initiated, and fluconazole was discontinued. A lumbar puncture was performed, and cerebrospinal fluid (CSF) studies did not suggest underlying CNS infection. CSF mycology cultures were ordered and no growth was observed at 21 days. Given positive serologies for *Toxoplasma* and radiologic findings on brain MRI, coupled with a negative *T. gondii* DNA PCR performed on CSF and the absence of neurological deficits on examination, only a presumptive diagnosis of cerebral toxoplasmosis could be made.

After two weeks of TMP/SMX for treatment of presumptive cerebral toxoplasmosis, a second brain MRI showed lesions of 0.7 cm on the same location as the first found (Image 3). Given the patient’s clinical and radiological improvement, she was discharged on B/FTC/TAF and TMP-SMX to complete a 6 week course of treatment for cerebral toxoplasmosis. The patient completed a 14 day course of liposomal amphotericin B while inpatient, and was discharged on itraconazole 200 mg three times daily as a loading dose, followed by maintenance therapy with itraconazole 200 mg twice daily for at least 12 months, in accordance with IDSA guidelines [7]. A follow-up appointment with the specialty immunology clinic was also scheduled.

The patient has been followed by the specialty immunology clinic, and her clinical status has improved significantly. She continues to take all her medications as prescribed for treatment of her HIV and OI prophylaxis, reports improvement in her energy levels, and has gained over 20 lbs. Her CD4 has also increased to 146 cells/mm^3^.

## Discussion

This case is significant as it represents the first reported instance in the U.S. of a patient with HIV/AIDS presenting with disseminated histoplasmosis-associated IRIS and cerebral toxoplasmosis co-infection. While IRIS associated with either *H. capsulatum* or *T. gondii* has been documented separately, the occurrence of these infections together is exceedingly uncommon. Prior to this case, such a co-infection associated with IRIS was reported only once in 2006 in Japan [8]. Prior studies have also demonstrated that histoplasmosis-associated IRIS with co-infection is rare. For example, *Melzani et al.* identified four cases of histoplasmosis co-infections with pathogens including CMV, MAC, and PJP in a 20 year case series, but no co-infection with toxoplasmosis was reported as part of the study, making our case particularly novel [5]. Our patient did not have clinical or imaging findings suggestive of toxoplasmosis-associated IRIS, supporting the diagnosis of histoplasmosis associated-IRIS with toxoplasmosis co-infection.

Individuals with HIV/AIDS are particularly susceptible to disseminated histoplasmosis, especially in regions where *H. capsulatum* is endemic, such as Cuba [9]. This fungus thrives in warm climates and contaminated soils, conditions prevalent in the patient’s home region of Matanzas. The majority of reported cases of histoplasmosis in Cuba originate from Matanzas, underscoring the region’s heightened endemicity [10]. Environmental factors and poor sanitation in Matanzas likely contribute to the high prevalence of fungal infections, posing a significant risk of reactivation in immunocompromised individuals like our patient with HIV/AIDS.

Our patient presented with classic symptoms of disseminated histoplasmosis, including significant weight loss, night sweats, and fatigue. Histoplasmosis-associated IRIS commonly presents with lymphadenopathy following initiation of ART, which was also observed in this case [2]. In contrast, the case reported by *Murata et al.* did not present with lymphadenopathy or weight loss. Instead, the patient presented only with high fever after initiation of an ART regimen consisting of zidovudine, lamivudine and indinavir. The patient subsequently developed pneumonia complicated by acute respiratory distress syndrome, acute renal failure, and ultimately died [8]. The differences between our cases and that reported by *Murata et al.* highlight the broad spectrum of symptoms related to disseminated histoplasmosis associated-IRIS, ranging from classic manifestations, as observed in our patient, to “atypical” symptoms that can mask the diagnosis of IRIS.

These symptoms often emerge or worsen following the initiation of ART, fitting the clinical criteria for unmasking IRIS, with symptoms appearing three months post-ART initiation, aligning with the typical IRIS timeline. This highlights the critical need for careful monitoring for OIs in advanced HIV patients during the early stages of ART, when immune recovery may unmask latent infections.

The management of IRIS involves three key components: treatment of the underlying OIs, ART, and adjunctive therapies [11]. As seen in this patient, identifying the infective agent and initiating the appropriate treatment as early as possible is crucial. ART should be continued in the absence of contraindications, given the risk of further complications due to ongoing immunosuppression [12]. In this case, ART initiation was delayed due to concerns about the risk of meningeal tuberculosis, requiring careful evaluation of the risks and benefits. Although IRIS is typically self-limited, ART is essential for restoring immune function and facilitating recovery. Adjunctive therapies, such as glucocorticoids or localized drainage, may be considered depending on the severity of the symptoms and the type of infection, though they were not necessary in this case.

Cerebral toxoplasmosis is the most frequent CNS opportunistic infection in HIV/AIDS patients, usually due to the reactivation of a latent infection [13]. This patient’s presumptive diagnosis was made by positive IgG anti-Toxoplasma antibodies and characteristic ring-enhancing lesions on brain imaging. Despite these findings, the patient exhibited no significant neurological deficits, which is atypical. Possible explanations include the early detection and treatment of cerebral toxoplasmosis following ART initiation, which might have mitigated neurological impact.

## Conclusion

Histoplasmosis, a systemic endemic mycosis, has been described as an underdiagnosed and neglected illness in the Americas [14]. It is caused by *H. capsulatum var. duboisii*, which is endemic to Central and West Africa, and *H. capsulatum var. capsulatum*, endemic to the Americas and is the focus of this case report [14, 15]. *Histoplasma* is a dimorphic fungus that is acquired via inhalation of microconidia aerosolized from environmental reservoirs, mainly soil containing bird and bat excreta [15]. Though most individuals exposed to *Histoplasma* spp. remain asymptomatic, individuals with HIV may develop disseminated histoplasmosis due to their impaired immune response. The most common reason for histoplasmosis in non-endemic regions is prior exposure to *H. capsulatum* in an endemic region and subsequent reactivation [16]. The patient described immigrated from Cuba where the warm climate, poor sanitary conditions, and endemicity of *H. capsulatum* likely contributed to her clinical presentation. Reviewing patient social history and understanding the potential exposures in certain geographic areas is crucial to developing a wide differential diagnosis and improving patients outcomes.

The patient described had also been diagnosed with presumptive cerebral toxoplasmosis, based on her brain imaging findings coupled with her serologies. Although the patient did not exhibit neurological deficits on exam and *Toxoplasma* DNA was not detected in her CSF via PCR, the decision was made to treat for cerebral toxoplasmosis according to IDSA guidelines, based on her clinical status and the understanding that *Toxoplasma* is the most common OI in HIV/AIDS. Toxoplasmosis is caused by the protozoan parasite *T. gondii*, which is acquired by several methods including: direct contact with cat litter, consumption of water or food contaminated by sporulated oocysts, and consumption of undercooked meat harboring tissue cyst of the parasite [17]. This clinical decision demonstrates the importance of balancing clinical presentation with diagnostic findings, especially in patients with HIV/AIDS, where multiple opportunistic infections can present simultaneously.

This case underscores the complexities and challenges in managing OIs during immune recovery in HIV/AIDS patients on ART, emphasizing the need for vigilant clinical monitoring and comprehensive diagnostic strategies. Continuous vigilant monitoring and a broad differential diagnosis are necessary in HIV/AIDS patients on ART, as rare co-infections can significantly impact patient outcomes.

## Appendix

**Fig. 1:**
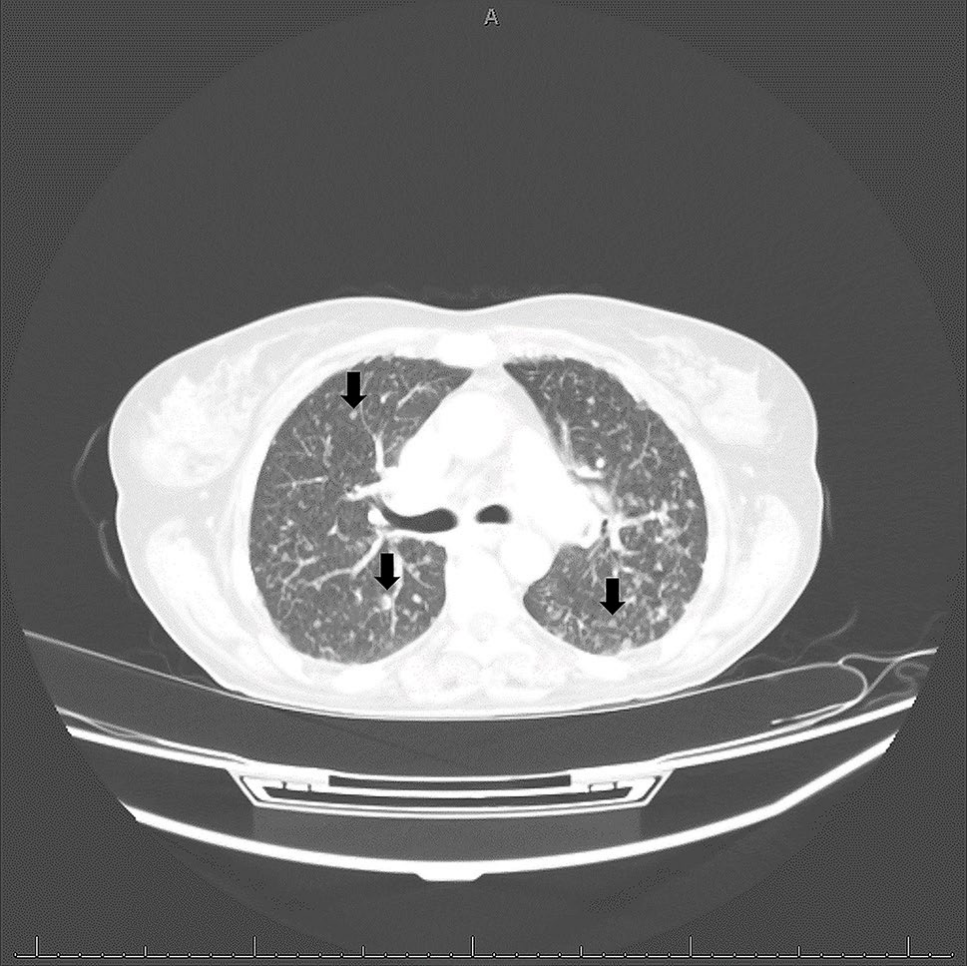
Chest CT showing innumerable scattered subcentimeter nodules throughout the bilateral lungs with associated mediastinal lymphadenopathy and biapical emphysematous changes

**Fig. 2:**
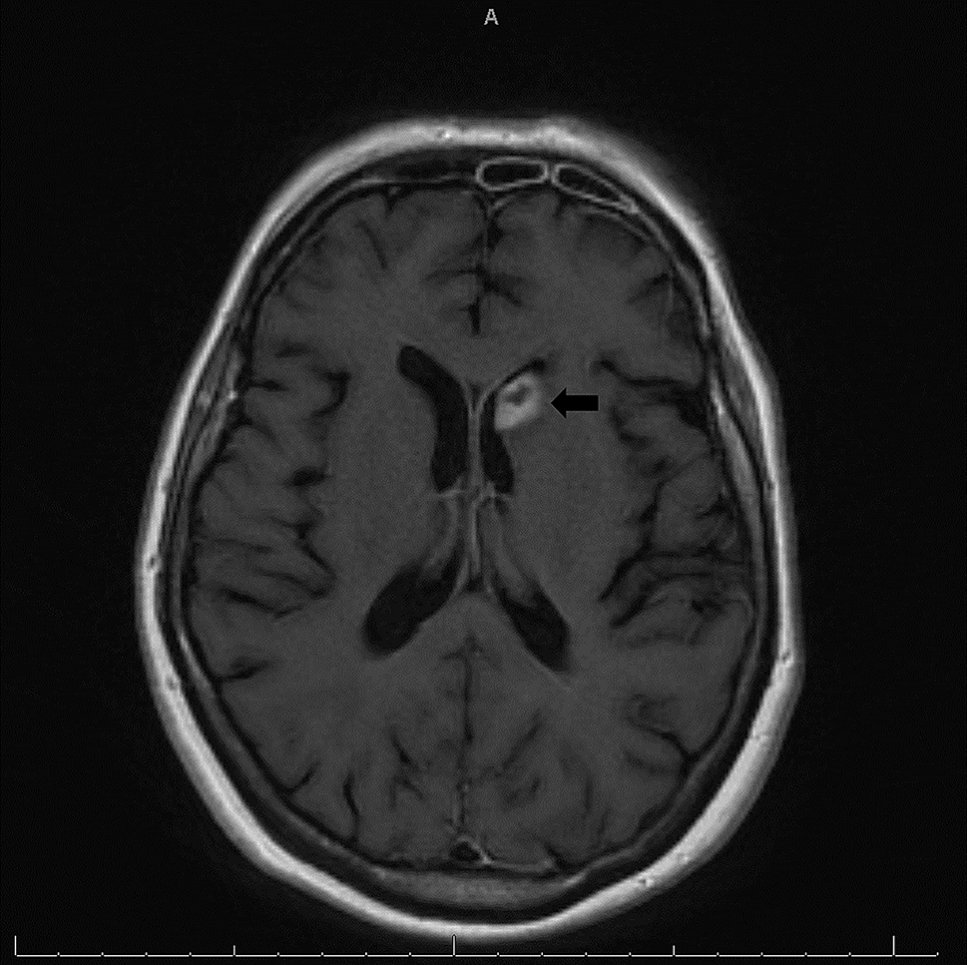
T1-weighted magnetic resonance image (MRI) with contrast medium showing a 1.6 cm rim enhancing lesion in the left caudate with surrounding edema and probable hemorrhagic components

**Fig. 3:**
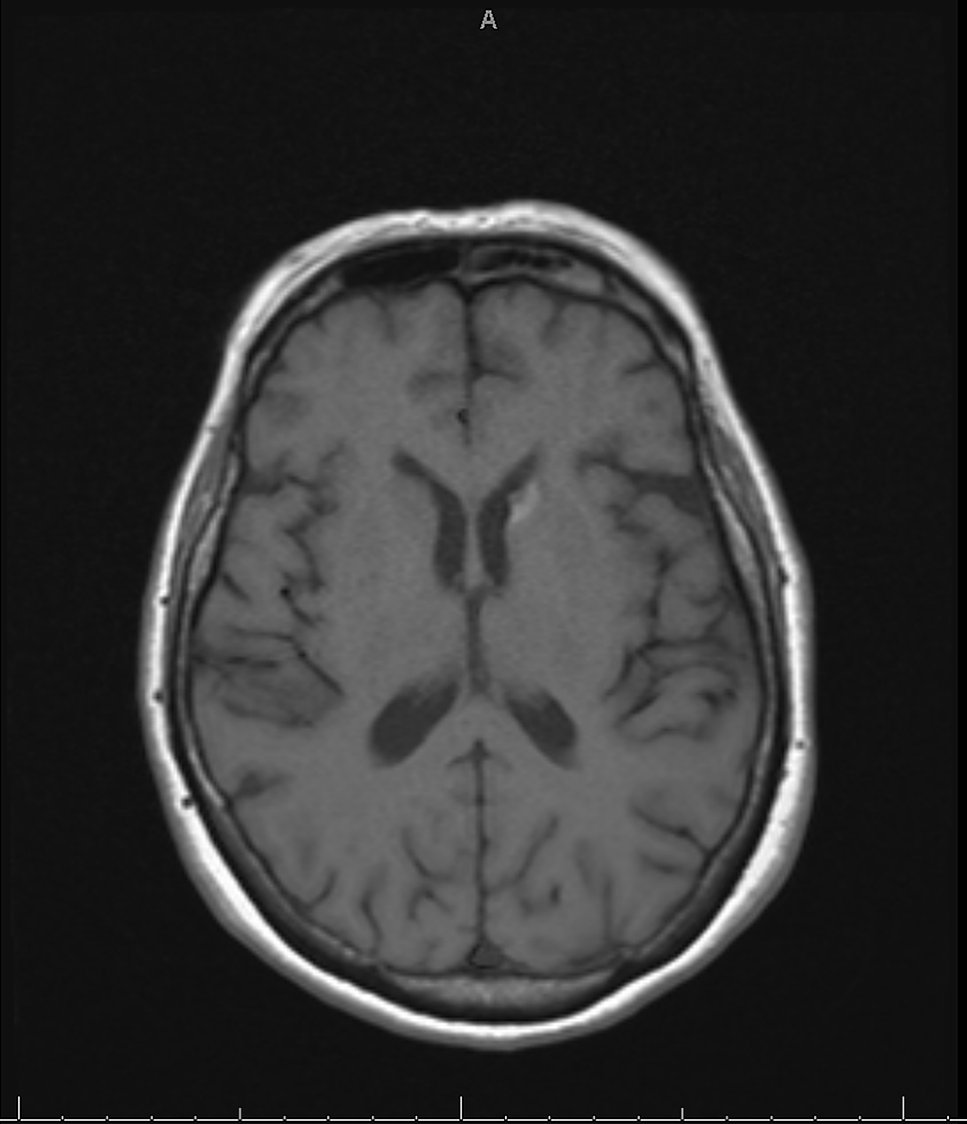
The T1-weighted magnetic resonance image (MRI) shows a reduced mass to 0.7 cm after 2 weeks of therapy for toxoplasmosis

## Data Availability

The datasets used and/or analysed during the current study are available from the corresponding author on reasonable request.

## Abbreviations

AFB: Acid-fast bacilli
ADRS: Acute respiratory distress syndrome
ART: Antiretroviral therapy
BAL: Bronchoalveolar lavage
B/FTC/TAF: Bictegravir/emtricitabine/tenofovir alafenamide
CMV: Cytomegalovirus
CNS: Central nervous system
CT: Computed tomography
HIV/AIDS: Human Immunodeficiency Virus/Acquired Immunodeficiency Syndrome
IGG: Immunoglobulin G
IRIS: Immune Reconstitution Inflammatory Syndrome
MAC: Mycobacterium avium complex
MRI: Magnetic Resonance Imaging
OIs: Opportunistic infections
PJP: Pneumocystis jirovecii pneumonia
RBC: Red blood cells
TMP-SMX: Trimethoprim/sulfamethoxazole
U.S.: United States
WBC: White blood cells
